# Development and Resolution of Secondary Adrenal Insufficiency after an Intra-Articular Steroid Injection

**DOI:** 10.1155/2022/4798466

**Published:** 2022-12-22

**Authors:** Jia Wei Tan, Sachin K. Majumdar

**Affiliations:** ^1^Department of Internal Medicine, Bridgeport Hospital, Yale New Haven Health System, Bridgeport, Connecticut, USA; ^2^Department of Endocrinology, Bridgeport Hospital, Yale New Haven Health System, Bridgeport, Connecticut, USA

## Abstract

Corticosteroid injections are commonly indicated in inflammatory conditions involving the soft tissues, tendon sheaths, bursae, and joints. Local corticosteroids carry a lower risk of complications than systemic corticosteroid but may be systemically absorbed and subsequently suppress the hypothalamic-pituitary-adrenal (HPA) axis. This can cause secondary adrenal insufficiency (SAI) as well as iatrogenic Cushing's syndrome. We report a 78-year-old female who presented with nonspecific gastrointestinal symptoms after a recent intra-articular steroid injection in her shoulder. She had hyponatremia, low morning cortisol, and failed to respond to high-dose cosyntropin. Further workup revealed the underlying cause to be SAI. Follow-up testing revealed a recovery of HPA responsiveness within 2 weeks of her initial diagnosis. *Conclusion*. Our case highlights how the hypothalamic-pituitary axis (HPA) can be suppressed with intra-articular steroids. The threshold to test corticosteroid users for adrenal insufficiency should be low in clinical practice, especially for those patients with nonspecific symptoms after steroid injections. Once diagnosed, temporary treatment with steroids may be required.

## 1. Introduction

One of the most common adverse effects of corticosteroid administration is secondary adrenal insufficiency (SAI) from suppression of the hypothalamic-pituitary-adrenal axis (HPA) by negative feedback. Oral and inhaled corticosteroids uses are known to cause SAI, whereas its occurrence with injectable corticosteroids is less common [1]. Local corticosteroids in the forms of inhaled, dermal, and intra-articular were developed to avoid first-pass hepatic metabolism and systemic side effects. However, studies have shown that systemic absorption and complications can occur with the use of local corticosteroids regardless of the treatment route [1]. When adrenal insufficiency is diagnosed, the HPA axis can take weeks to recover, and hence, patients may require glucocorticoid replacement therapy until full recovery of adrenal function [2, 3]. Herein, we report a 78-year-old woman with adrenal insufficiency after an intra-articular corticosteroid injection who subsequently demonstrated HPA recovery within 2 weeks of her initial diagnosis.

## 2. Case Report

A 78-year-old female presented to the emergency department with nausea, abdominal pain, and fatigue for three to four days. The abdominal pain was diffuse, nonremitting, and sharp in nature. It was not associated with food intake. She denied any fever, vomiting, or altered bowel habits. She received one dose of intra-articular cortisone into her left wrist 1 week prior to admission for the treatment of carpal tunnel syndrome (CTS). She also received one dose of intra-articular steroid injection in her right shoulder 2 months prior to presentation. She reported that her blood glucose levels were elevated after the steroid injection. She denied dizziness, salt craving, hypoglycemic episodes, tanning of skin or mucous membranes, or loss of weight. She has a past medical history of type 2 diabetes mellitus, insomnia, and gastroesophageal reflux disease. Medications include gabapentin, insulin, and mirtazapine.

On admission, her blood pressure was 115/69 mmHg, heart rate 68 beats/min, and she was not orthostatic. Her body mass index (BMI) was 21.03 kg/m^2^. She had mild diffuse abdominal tenderness without guarding or rigidity. Blood chemistry revealed a serum sodium of 122 mmol/L (ref: 135–145 mmol/l), 126 mmol/L corrected for glucose, chloride of 87 mmol/L (ref: 98–107 mmol/L), potassium of 5.2 mmol/L (ref: 3.3–5.3 mmol/L), and glucose of 275 mg/dL. Complete blood count showed hemoglobin of 12.9 g/dL (ref 11.7–15.5 g/dL), hematocrit of 35% (ref: 35–45%), and WBC count of 5.8 × 10003/*μ*L (ref: 4.0–11.0 × 1000/*μ*L). Further tests revealed a serum plasma osmolality of 277 mOsm/kg (ref: 275–295 mOsm/kg), urine osmolality of 240 mOsm/kg (ref: 50–1200 mOsm/kg), urine sodium of 55 mmol/L, urine potassium of 9 mmol/L, and serum creatinine of 0.7 mg/dL (ref: 0.40–1.30 mg/dL). Her findings were consistent with euvolemic hypotonic hyponatremia.

A morning cortisol level for this patient was less than 0.5 *μ*g/dL at 6:18 am (ref: Cortisol AM: 6.0–18.4 *μ*g/dL). A high-dose cosyntropin stimulation test was then performed.  Table 1 summarizes the findings for initial cortisol and the high-dose (250 mcg) cosyntropin test which revealed subnormal response to cosyntropin and relatively low ACTH. She was diagnosed with secondary adrenal insufficiency (SAI). The rest of the biochemical analysis, including thyroid-stimulating hormone (TSH), calcium, phosphorus, total protein albumin, lipid panel, and globulin, was within the normal range. Additional tests to evaluate adrenal function revealed an ACTH level of 8.7 pg/dl at 4.30 p.m. (ref: 7.2–63.3 pg/mL (a.m. draws), aldosterone <1 ng/dl (ref: supine 8:00–10:00 am 3–16 ng/dL), and plasma renin activity of 1.13 ng/mL/h (ref: 0.25–5.82 ng/mL/h). Her dehydroepiandrosterone sulfate (DHEA-S) was 15.1 *μ*g/dL (ref: 35.0–430.0 *μ*g/dL). Prolactin was 16.2 ng/mL (ref: 4.8–23.3 ng/mL), follicle-stimulating hormone was 56.6 (ref: Post-Menopausal Females: 25.8–134.8 mIU/mL), and luteinizing hormone was 33.1 mIU/mL (ref: Post-Menopausal Females: 7.7–58.5 mIU/mL). Pituitary magnetic resonance imaging (MRI) showed a 3-4-millimeter lesion in the pituitary consistent with a microadenoma. Endocrinology was consulted, and it was deemed unlikely that the microadenoma was causing adrenal suppression. The patient remained hemodynamically stable and her symptoms improved during hospitalization. Her hyponatremia and abdominal pain resolved with supportive management which include fluid restriction (1.5 liters per day) and correction of hyperglycemia. She was discharged from the hospital without steroid replacement.

One week after hospitalization, she was seen in the outpatient clinic of endocrinology and was doing well. However, she was admitted to the hospital the next day for acute gastrointestinal symptoms and was found to have a serum sodium of 118 mmol/L (ref: 136–144 mmol/L). Her random cortisol level drawn at 5:13 AM was 16.4 mcg/dL (ref: Cortisol AM: 6.0–18.4 *μ*g/dL), 8 days after she had initially failed cosyntropin stimulation testing. Because her admitting physicians were aware of her recent diagnosis of adrenal insufficiency, she was placed on empiric hydrocortisone until she could be more completely evaluated. Once her cortisol level returned and she was reassessed clinically, hydrocortisone was discontinued and repeat stimulation testing revealed a normal response to cosyntropin (Table 2). Her symptoms were subsequently found to be primarily gastrointestinal in nature, and she revealed a history of copious water consumption at home. Therefore, her hyponatremia was better explained by nonosmotic antidiuretic hormone release due to nausea and gastrointestinal distress in combination with excess water consumption, and her adrenal insufficiency was considered transient and noncontributory to her second admission. However, concerns remained as to whether she should have been given a brief course of empiric steroids after her first admission.

## 3. Discussion

We report a 78-year-old woman presenting with hypotonic hyponatremia complicated by transient adrenal insufficiency most likely caused by recent intra-articular steroid injections of the wrist and shoulder. Her serum osmolality and urine sodium were elevated, indicating the effect of antidiuretic hormone (ADH). She appeared euvolemic and had normal orthostatic vital signs, thus narrowing the differential diagnosis to syndrome of inappropriate antidiuresis (SIAD), secondary adrenal insufficiency (SAI), hypothyroidism, and decreased intake of solutes (e.g., beer potomania, tea-and-toast diet). The patient was on mirtazapine, which was continued throughout hospitalization; hence, it is possible that there was a component of mirtazapine-induced SIAD. She appeared well nourished and lacked features of severe hypothyroidism, and her serum albumin and TSH were in normal ranges. Given her history of intra-articular steroid injections, serum cortisol and cosyntropin testing were ordered revealing evidence of SAI. Her serum aldosterone level was low, raising the possibility of primary adrenal insufficiency (PAI). However, her clinical picture is inconsistent with PAI as her blood pressure was mildly elevated, and her serum potassium and acid-base status were normal. She was on losartan which may possibly explain her low aldosterone level. Her ACTH and DHEA-S were low, consistent with SAI.

SAI is the most common form of adrenal insufficiency, and it is commonly seen with exogenous steroid administration and their subsequent withdrawal [5]. Cortisol exhibits negative feedback on the release of corticotropin-releasing hormone (CRH), ACTH, and ADH [6]. Exogenous corticosteroids can result in suppression of the HPA axis that may last after the offending agent is discontinued, and since cortisol levels may remain low, the usual suppressive effect on ADH may also be absent. Therefore, ADH levels can be elevated in adrenal insufficiency resulting in diminished free water excretion and predisposition to hyponatremia, particularly when water is consumed in excess, as occurred with our patient. Cortisol secretion is diurnal, and a low morning cortisol level is indicative of adrenal insufficiency. However, levels at other times lack diagnostic utility and might be difficult to interpret on their own. An ACTH level would differentiate between primary and secondary adrenal insufficiency [7]. A low-normal ACTH points towards SAI and warrants an MRI of the hypothalamic-pituitary area to rule out hypothalamic or pituitary lesions such as craniopharyngioma, metastasis, and pituitary adenoma [8]. DHEA-S, which is an ACTH-responsive hormone, has a long half-life (about 20 h) and less diurnal variation than cortisol [9] and can serve as an adjunct measure in the diagnosis of adrenal insufficiency. A middle to upper range of normal reference value of DHEA-S indicates that the underlying pathology is less likely due to SAI [7].

Interestingly, she revealed evidence of HPA recovery on a subsequent admission one week later. This highlights how the transient suppression of the HPA axis by exogenous steroids can complicate the diagnosis and management of steroid-associated adrenal insufficiency since the degree of morbidity caused and the time course of full HPA recovery are unclear. The distinction between transient HPA suppression and adrenal insufficiency is challenging and may confound the evaluation of patients when they present with features such as hyponatremia, hypotension, and hypoglycemia since they can indicate adrenal insufficiency as well as alternative diagnoses.

In our case, we found that patient underwent a local corticosteroid injection for CTS. There is no published literature on the association of adrenal insufficiency with a local steroid injection for CTS to date. A randomized clinical trial with a 5-yearfollow-up reported improvement in quality of life among CTS patients who received local corticosteroid injection and did not observe adverse effects of local steroid injection [10]. Therefore, we postulate that the culprit was the intra-articular corticosteroid injection that the patient received two months prior to admission but cannot exclude an effect of the recent one for CTS either.

The incidence of adrenal insufficiency is higher with intra-articular corticosteroid injections than with oral corticosteroids [52.2% (95% CI, 40.5–63.6) vs 48.7% (95% CI, 36.9–60.6)]. SAI has been reported after local corticosteroid injections into various locations including the greater trochanter, epidural space, knee, and ankle [11]. After an intra-articular triamcinolone injection, a significant decrease in serum cortisol and urine cortisol excretion was seen [12]. Postinjection, the serum cortisol was nadired in 24 hours and normalised in 7 to 14 days [12]. Duclos et al. found that 90% of the patients had adrenal insufficiency 2 days postintra/periarticular glucocorticoid injection, and serum cortisol remained suppressed 7 and 14 days postinjection [11]. They have also found that the degree of adrenal suppression was directly proportional to the steroid dose injected. Habib et al. found that 25% of patients who received an intra-articular corticosteroid injection for the knee joint [80 mg of methylprednisolone acetate (MPA)] had adrenal suppression for at least 2 to 4 weeks postinjection, and the adrenal function recovered in all patients by week 8 [13]. Interestingly, none of the adrenal insufficiency patients were symptomatic. The group subsequently conducted a study using 6 mg of betamethasone acetate/betamethasone sodium phosphate and found that only 0.05% of the patients who received intra-articular corticosteroid injections had SAI 3 weeks postinjection [14]. Eighty mg of MPA is approximately equivalent to 15 mg of betamethasone, suggesting that the systemic absorption of steroids and subsequent risk of SAI are likely dose-dependent.

Although HPA suppression typically lasts for weeks, the duration could be prolonged to 9 months [1]. This could be due to atrophy of the zona fasciculata and reticularis with chronic ACTH deficiency resulting in a decrease production of cortisol [15]. Given some uncertainty exists about the duration of suppression, education regarding symptoms and management of adrenal insufficiency should be given to patients who are vulnerable to adrenal crises in times of stress or illness while the HPA is suppressed.

There are no specific guidelines concerning the monitoring and evaluation of the HPA axis for recovery in the setting of SAI related to intra-articular glucocorticoid injections. Most cases are dealt with on an individual basis depending on the patient's treatment plan and most likely go undiagnosed and unnoticed. If SAI is suspected, a cosyntropin stimulation test can be performed to evaluate HPA axis recovery. Situations, where it would be reasonable to perform testing, would be in the setting of symptoms concerning for adrenal insufficiency within three months of a corticosteroid injection, in the setting of anticipated stressors such as planned surgery, or acute stressors such as illness or hospitalization, and if there is a concern for suppression prior to repeated intra-articular steroid injections. The insulin tolerance test (ITT) is another dynamic test that assesses HPA integrity. This test is contraindicated in cardiac dysrhythmias patients and requires close supervision [16]. In our case, ITT was deemed unsafe to perform on this elderly lady with multiple comorbidities who were acutely ill, especially since we had access to the cosyntropin stimulation test which is simpler and less invasive than the ITT.

The high-dose (250 *μ*g of corticotrophin) ACTH stimulation test is supra-physiological and may miss mild forms of adrenal insufficiency, and the low-dose (1 *μ*g of corticotrophin) ACTH stimulation testing gives results that are comparable or even more sensitive than that of high-dose testing [17, 18]. Ospina et al. found that both high- and low-dose stimulation tests had similar diagnostic accuracy in SAI. For the high-dose stimulation test, the sensitivity is 0.64 (95% CI 0.52–0.73), and the specificity is 0.93 (95% CI 0.89–0.96). For the low-dose stimulation test, the sensitivity is 0.83 (95% CI 0.75–0.89), and the specificity is 0.86 (95% CI 0.78–0.91) [19]. However, low-dose testing has not been validated in patients with acute illnesses, hypoalbuminemia, or acute hypothalamic-pituitary disorders. Furthermore, there are no commercially available preparations for the low-dose cosyntropin. There is a possibility of misdiagnosis and inconsistency in dosing due to errors in preparation since 250 mcg needs to be diluted to 1 mcg by the pharmacy on demand. Therefore, the low-dose ACTH testing is not recommended as first-line testing for adrenal insufficiency.

In conclusion, we describe a patient with transient SAI in the setting of recent corticosteroid injections who had a quick recovery of her HPA axis; however, her course was complicated by uncertainty as to how much adrenal impairment contributed to her presentation. This case illustrates the dilemma of managing patients with low cortisol levels in the weeks to months following corticosteroid injections and highlights the need for more detailed studies and clinical guidelines.

## Figures and Tables

**Table 1:** Initial admission cosyntropin stimulation test.

	ACTH level [7.2–63.3 pg/mL (a.m. draws)]	AM cortisol (6.0–18.4 *μ*g/dL)	After 30 minutes (*μ*g/dL)	After 60 minutes (*μ*g/dL)	The next day AM ACTH (7.2–63.3 pg/mL)
Baseline	8.7	<0.5			1.6
Cosyntropin stimulation			5.3	7.7	

**Table 2:** Second admission (8 days after initial cosyntropin testing) cosyntropin stimulation test.

	ACTH level^*∗*^[7.2–63.3 pg/mL (a.m. draws)]	AM cortisol^*∗*^(6.0–18.4 *μ*g/dL)	After 30 minutes^*∗∗*^ (*μ*g/dL)	After 90 minutes^*∗∗*^ (*μ*g/dL)
Baseline	14.4	16.4		
Cosyntropin stimulation			8.1	14.5

^*∗*^8 days after initial cosyntropin stimulation test. ^*∗∗*^11 days after initial cosyntropin test, blood drawn late so second result is at 90 minutes. Our laboratory uses the Roche Diagnostics Elecsys® cortisol II assay which results in cortisol values approximately 30% lower than prior assays. Previously a value of >18 mcg/dL was considered normal, but with the assay in use a value of >13.5 mcg/dL was adopted as a normal response [4].

## Data Availability

Data sharing is not applicable to this article as no new data were created or analyzed in this study.

## Abbreviations

HPA: Hypothalamic-pituitary-adrenal axis
SAI: Secondary adrenal insufficiency
PAI: Primary adrenal insufficiency.
